# Expressive suppression and enhancement during music-elicited emotions in younger and older adults

**DOI:** 10.3389/fnagi.2015.00011

**Published:** 2015-02-18

**Authors:** Sandrine Vieillard, Jonathan Harm, Emmanuel Bigand

**Affiliations:** ^1^Laboratory of Psychology, Psychology, Université de Franche-ComtéBesançon, France; ^2^Psychology, LEAD-CNRS, Psychology, Université de BourgogneDijon, France

**Keywords:** musical emotions, aging, expressive suppression, expressive enhancement, physiological measures

## Abstract

When presented with emotional visual scenes, older adults have been found to be equally capable to regulate emotion expression as younger adults, corroborating the view that emotion regulation skills are maintained or even improved in later adulthood. However, the possibility that gaze direction might help achieve an emotion control goal has not been taken into account, raising the question whether the effortful processing of expressive regulation is really spared from the general age-related decline. Since it does not allow perceptual attention to be redirected away from the emotional source, music provides a useful way to address this question. In the present study, affective, behavioral, and physiological consequences of free expression of emotion, expressive suppression and expressive enhancement were measured in 31 younger and 30 older adults while they listened to positive and negative musical excerpts. The main results indicated that compared to younger adults, older adults reported experiencing less emotional intensity in response to negative music during the free expression of emotion condition. No age difference was found in the ability to amplify or reduce emotional expressions. However, an age-related decline in the ability to reduce the intensity of emotional state and an age-related increase in physiological reactivity were found when participants were instructed to suppress negative expression. Taken together, the current data support previous findings suggesting an age-related change in response to music. They also corroborate the observation that older adults are as efficient as younger adults at controlling behavioral expression. But most importantly, they suggest that when faced with auditory sources of negative emotion, older age does not always confer a better ability to regulate emotions.

## Introduction

A growing body of empirical research has explored the age-related changes in the ability to regulate emotions, suggesting a developmental gain in this domain. For instance, compared to younger adults, older people report to be more successful in controlling their emotions (Lawton et al., 1992; Gross et al., 1997; Phillips et al., 2006), have better control of negative emotions (Lawton et al., 1992; Gross et al., 1997), were quicker to return to more positive states after a negative mood state (Carstensen et al., 2000; Larcom and Isaacowitz, 2009) and use more efficient emotion regulation strategies to deal with interpersonal conflicts (Blanchard-Fields et al., 2004). Some of these findings have been interpreted within the framework of Socioemotional Selectivity Theory (Carstensen et al., 1999) in terms of motivational changes, arguing that perceived constraints on time left to live activate effortful to prioritize the optimization of emotional experience in later life. These findings are somewhat surprising because they suggest that despite the physical and cognitive declines associated with the normal aging process, the ability to control emotions and to maintain well-being are spared in old age. Recently, additional findings have suggested that, compared to their younger counterparts, older adults need fewer cognitive resources to effectively down-regulate negative emotional state in response to disgust-inducing film clips (Scheibe and Blanchard-Fields, 2009) or implement expressive suppression while looking at negative and positive pictures (Emery and Hess, 2011). Although the exact mechanisms behind these effects remain unexplained, these data have been interpreted as evidence that the regulation of inner state and outward emotional expression may be less costly in older age.

In the literature on emotion regulation, several forms of regulation strategies have been distinguished. Reappraisal, defined as the aim to change an emotional response by reinterpreting the meaning of the emotional event, and expressive suppression, defined as the aim to hide expressive behavioral cues, represent two distinct strategies (Gross, 1998; Gross and John, 2003) and are recognized as the most studied forms of regulation. Among them, expressive suppression, and to a lesser extent, its opposite form, enhancement or amplification, are commonly used in an attempt to change certain components and processes of the emotional response (Gross et al., 2006). Whereas expressive regulation has been found to be cognitively costly, reappraisal has not (Gross, 2002). The expressive regulation unfolds once the emotion is elicited. Consequently, it comes later in the emotion generative process, is associated with a continual self-regulatory effort and thus represents a form of regulation particularly costly in cognitive resources.

Paradoxically, instructing to suppress facial expression in response to films portraying injustice, Phillips et al. (2008) found that younger and older adults were equally able to regulate their outward expressions of emotion. The preservation of expressive suppression in older adults was still found in response to disgusting and sad film (Shiota and Levenson, 2009) supporting the hypothesis of an age-related stability in behavioral suppression. In a research by Kunzmann et al. (2005), younger and older adults were asked to watch disgusting films while amplifying or suppressing their facial expressions. They found no difference between age groups in their emotional and physiological responses to expressive regulation, suggesting that suppression and enhancement are spared from age-related losses, at least when it comes to negative emotions. In this research, it is noteworthy that the emotional stimuli were found to be stronger for younger adults than their older counterparts, rendering expressive regulation potentially easier for older people. More generally, taking into account that expressive regulation is more costly than the other emotion regulation strategies (Gross, 2002), the preserved expressive regulation skills in older adults compared to younger adults suggests that different strategies were used across age groups to control outward signs of emotions.

Among the potential strategies at play, one might suspect that when faced with visual negative scenes, older adults try to reduce the emotional impact of the stimuli by deploying their attention away from them. In line with this hypothesis, previous works have shown that older adults tend to deploy their visual attention away from negative stimuli (Isaacowitz et al., 2006). Other findings have shown that when controlling gaze direction, older people were less successful than younger adults to regulate unpleasant emotion elicited by a visual scene (Opitz et al., 2012). Such results raise the possibility that attentional deployment might partly explain successful emotion regulation in older adults. In this respect, we wanted to neutralize the attention redeployment strategy by means of emotional stimuli that do not allow participants to reallocate attention away from the emotional source. For this purpose, we used musical material known to be a powerful source of emotion elicitation (e.g., Juslin and Sloboda, 2001). Unlike many prior studies examining age differences in emotion regulatory success that used stimuli not designed to induce affective state *per se*, current musical stimuli was selected to evoke strong emotional responses. Furthermore, very few studies have investigated older adults' abilities to both suppress and enhance expressions elicited by positive and negative emotions in the same experiment. Thus, our purpose was to extend previous findings by addressing whether expressive regulation may lead to a general shift toward strength or weak emotional response depending on the hedonic valence of musical emotion. Age-related differences in emotional processing have typically been found on the valence dimension, it is thus likely that the impact of age on regulation skills varies as a function of the positive or negative valence of the musical stimuli. As stressed above, enhancement and suppression have been shown to be the most cognitively costly forms of emotion regulation (Richards and Gross, 2000; Robinson and Demaree, 2009). Therefore, we focused on these forms to test whether the declines of older adults' executive functioning would result in greater difficulty to inhibit or exaggerate the expression of positive and negative emotions elicited by musical excerpts.

In the literature on emotion, there is general agreement that an emotional response involves several changes that occur simultaneously in the subjective, behavioral, and physiological response systems. Given that prior findings have indicated that these response systems may show different age trajectories (e.g., Kunzmann et al., 2005), a whole picture of the age-related differences in expression regulation abilities needs investigations that take into account the multidimensional nature of emotion. Accordingly, we indexed the difficulty to implement expressive regulation by examining the affective, behavioral, and physiological responses of younger and older participants. Affective response was assessed using subjective ratings of emotional state (valence and arousal), engagement in music listening, and reappraisal strategy. Behavioral response was evaluated using facial muscle activity from the *corrugator supercilli* and the *zygomaticus major* areas. These EMG activities were also recorded to provide manipulation check. Physiological reactivity was indexed by electrodermal (Skin Conductance Level) and cardiac (Heart Rate, Systolic Blood Pressure, Diastolic Blood Pressure) activity. We applied a within-subjects design to yield for a direct comparison of affective, behavioral and physiological patterns associated with different emotion regulation in participants who each served as their own control. We included three conditions of emotion regulation (i.e., free expression of emotion, expressive enhancement, expressive suppression) to explore their effects when employed on both positive and negative emotions elicited by music. In addition to the valence and intensity assessment of the affective response, we decided to investigate whether when instructed to suppress or exaggerate their behavioral expression, participants would simultaneously use cognitive strategies to regulate their inner feelings.

Based on previous findings, no age-related difference in the ability to suppress or exaggerate positive and negative expression should *a priori* be expected. However, there is a possibility that a musical context requires the use of more costly regulation strategies than a simply redirection of attention from the visual scene. Therefore, due to their limited cognitive resources, older adults were expected to display more difficulties to voluntary suppress the expression of emotions than their younger counterparts. This leads to the prediction that in the condition of expressive suppression, and compared to younger adults, older adults should show similar ability to deliberately suppress the expression of emotions but they should display more difficulties in terms of affective (e.g., lesser impact on emotional experience) and physiological (e.g., higher physiological activation) consequences. Moreover, because aging is associated with a reduced physiological flexibility and a prolonged physiological arousal (Charles, 2010), we predicted that physiological consequences in older adults should be more prominent during the (recovery) period following the musical excerpt presentation. A number of authors have speculated about the possibility that older adults' preference for positive over negative information in cognitive processes (i.e., called positivity effect) may serve their purpose of maintaining well-being. But up to date, the causal link between positivity effect and emotion regulation outcomes in older adults has not been convincingly established (Isaacowitz and Blanchard-Fields, 2012; Reed and Carstensen, 2012). In this context, we addressed positivity effect and emotion regulation processes separately. Regarding the positivity effect, several findings have suggested that its emergence would depend on the amount of cognitive control required by the task, pointing out that experimental conditions allowing participants to have greater focus on their emotional state can facilitate the emergence of positivity effects in aging (e.g., Kennedy et al., 2004; Yang and Ornstein, 2011; Tomaszczyk and Fernandes, 2012). More generally and as stated by the cognitive control account of the SST model, the positivity effect is most likely to be found when information processing is not constrained (Reed and Carstensen, 2012). In this respect, it was expected that the free expression emotion condition that does not constraint information processing would be the most appropriate test for positivity-related prediction. In line with the appraisal theory, it is assumed that the subjective evaluation of emotional stimuli is cognitive in nature. Consequently, it is expected that participants' self-reported emotional state following positive and negative musical stimuli may be a relevant cue to capture age-related changes in emotional bias.

## Methods

### Participants

Sixty-one native French-speaking volunteers (16% amateur musicians) participated in the current study. The sample was divided into two age groups, with 31 younger adults (20–45 years; *M* = 31; 52% females), and 30 older adults (50–78 years; *M* = 64; 66% females), such as that age ranges were quite similar across cohorts. All participants reported no neurological or psychiatric antecedent. In older adults, Mini-Mental State Examination (MMSE; Folstein et al., 1975) scores varied from 27 to 30 (*M* = 29). Participants were recruited at several departments of the University of Franche-Comté and through senior social programs in Besançon. All participants received course credit or financial compensation for participating. As illustrated Table 1, older adults did not report statistically different levels of education, health, amateurish musical practice, or number of hours devoted to music listening per week in comparison with younger adults. However, older adults reported an almost statistically significant difference in frequency of classical music listening in comparison with younger adults. The examination of affective functioning indicated that older and younger adults did not significantly differ on depression (BDI-II; Beck et al., 1998), state anxiety (STAI-Y; Spielberger, 1993), trait anxiety (STAI-Y; Spielberger, 1993), PANAS positive affects (Watson et al., 1988), PANAS negative affects (Watson et al., 1988), BEQ positive expressivity, BEQ negative expressivity (Adapted from BEQ by Gross and John, 1997), and Reappraisal subscale of ERQ (Christophe et al., 2009) measures. As reported by previous findings (Nolen-Hoeksema and Aldao, 2011; Brummer et al., 2013), scores from the Suppression subscale of the ERQ (Christophe et al., 2009) showed that older adults reported using more expressive suppression as emotion regulation strategies then their younger counterparts. As stated by prior works showing age-related changes on the auditory system (e.g., Enrietto et al., 1999), the control of the auditory perception showed statistically significant difference of hearing thresholds (dB) between age groups for all the tested frequencies. In line with previous studies on fluid intelligence (e.g., Baltes et al., 1999) and cognitive flexibility (e.g., Schaie et al., 1991) in old age, the control of participants' cognitive functioning showed significant age-related differences on fluid intelligence (measured by Raven's progressive matrices, set I, Raven et al., 1998, updated 2003), and cognitive flexibility (measured by the Trial Making Test, Reitan, 1958). However, and inconsistency with previous works pointing out the impairment of executive function in the elderly, no significant difference was found between groups either on working memory (measured by letter-digit sequencing from WAIS-III, Wechsler, 2000) or inhibitory control (measured by IF index of the Victoria Stroop test), suggesting that the older adults of the current sample had relatively preserved executive function. Finally, and in agreement with previous findings on age-related differences in personality (e.g., Donnellan and Lucas, 2008), the examination of personality trait (measured by the French validation of NEO-P-IR by Plaisant et al., 2010) showed age-related differences on the mean scores of Agreeableness and Conscientiousness. No other significant difference was found.

**Table 1:** **Sample characteristics**.

	**Younger adults (*n*= 31)**	**Older adults (*n*= 30)**	**Levene's homogeneity test**	**Age group difference**
	**Mean**	**Mean**	***p*-value**	***p-*value^a^^,^^b^**
**DEMOGRAPHIC**				
Age (years)	31 (10.30)	64 (7.45)	0.00	0.00^b^
Education (years)	14 (2.02)	13 (3.02)	0.10	0.10^a^
Self-reported health (max.5)	4.34 (0.60)	4.02 (1.02)	0.04	0.29^b^
Musical practice (ratio)	0.23 (0.43)	0.10 (0.31)	0.01	0.19^b^
Frequency of classical music listening (ratio)	0.13 (0.34)	0.33 (0.48)	0.00	0.06^b^
Numbers of hours devoted to music listening per week	7.48 (8.33)	8.75 (6.73)	0.33	0.51^a^
**AUDITORY THRESHOLDS**				
500 Hz (dB)	18	23	0.08	0.01^a^
1000 Hz (dB)	10	26	0.03	0.00^b^
2000 Hz (dB)	19	37	0.17	0.02^a^
4000 Hz (dB)	24	37	0.12	0.00^a^
8000 Hz (dB)	18	35	0.01	0.00^b^
**COGNITIVE FUNCTIONING**				
Fluid intelligence: advanced progressive matrices (Set 1, max.12)	9.81 (1.78)	8.70 (2.24)	0.51	0.04^a^
Inhibition: victoria stroop (IF)	2.24 (1.95)	2.41 (0.89)	0.37	0.67^a^
Flexibilty: trail making test (s)	30.03 (27.56)	50.60 (23.83)	0.76	0.03^a^
Working memory: letter-number sequencing (max.21)	12.35 (2.58)	11.33 (2.52)	0.83	0.12^a^
**AFFECTIVE FUNCTIONING**				
BDI-II (max.63)	4.84 (3.35)	5.87 (4.72)	0.18	0.33^a^
STAI-Y anxiety state (max.80)	26.94 (7.39)	26.23 (4.40)	0.03	0.55^b^
STAI-Y anxiety trait (max.80)	35.71 (6.54)	36.23 (6.38)	0.75	0.75^a^
PANAS positive affect (max.50)	34.81 (5.68)	35.00 (6.45)	0.68	0.90^a^
PANAS negative affect (max.50)	16.13 (4.25)	16.98 (7.79)	0.01	0.50^b^
ERQ supression (max.28)	13.16 (6.27)	17.33 (6.32)	0.98	0.01^a^
ERQ reappraisal (max.42)	28.42 (5.82)	27.22 (7.15)	0.46	0.47^a^
BEQ negative expressivity (max.7)	4.47 (1.29)	4.60 (2.93)	0.49	0.82^a^
BEQ positive expressivity (max.7)	4.92 (1.25)	6.28 (5.15)	0.24	0.16^a^
BEQ global expressivity (max.7)	4.64 (1.02)	4.72 (0.70)	0.09	0.73^a^
**PERSONALITY TRAIT**				
NEO-PI-R neuroticism score (max.192)	86.74 (13.50)	82.93 (21.49)	0.22	0.41^a^
NEO-PI-R extraversion score (max.192)	107.19 (20.47)	109.66 (14.90)	0.20	0.59^a^
NEO-PI-R openness score (max.192)	112.39 (15.36)	117.60 (21.83)	0.27	0.28^a^
NEO-PI-R agreeableness score (max.192)	121.71 (20.68)	135.91 (18.16)	0.31	0.01^a^
NEO-PI-R conscientiousness score (max.192)	114.48 (20.13)	126.51 (17.85)	0.25	0.02^a^

*Standard deviations are listed in parantheses; ERQ, Emotion Regulation Questionnaire; PANAS, Positive and Negative Affect Schedule; NEO-PI-R, Revised NEO Personality Inventory; BEQ, Berkeley Expressivity Questionnaire*.

a *Comparisons were assessed by Student's t-test*.

b *Comparisons were assessed by Mann–Whitney's U-test*.

### Apparatus

Participants were tested individually in a quiet room at stable ambient temperature at the university. Physiological activity was monitored continuously during the listening and rating phases using an MP150 Biopac system (Biopac Systems, Inc., Goleta, CA) at a sampling rate of 500 Hz and processed using AcqKnowledge sofware. E-prime software (Schneider et al., 2002) was used for excerpt presentation and ratings recording. Musical excerpts were presented binaurally over the Professional 240 Sennheisser headphones.

### Materials

Twenty eight musical excerpts (including four stimuli for the training phase) were taken from two validated sets of unfamiliar musical excerpts (Bigand et al., 2005; Eerola and Vuoskoski, 2011) following the rules of the Western tonal system with the aim of conveying positive high arousing emotions and negative high arousing emotions, respectively. More specifically, 16 musical stimuli (including the four stimuli for the training phase taken from each of the two emotion categories) taken from Bigand et al. (2005)' battery were chosen to be representative of key musical periods of Western classical music (e.g., baroque, classical, romantic, and modern) and from the most important instrumental groups (solo, chamber music, orchestra). Twelve musical stimuli taken from Eerola and Vuoskoski (2011)' battery corresponded to unfamiliar film music composed for the purpose of mediating powerful emotional cues. Four additional neutral auditory stimuli consisting in tuning orchestra or playing scales in cello or piano were used as baseline condition in order to test whether affective, behavioral, and physiological responses to such stimuli varied as a function of age groups. One peaceful musical excerpt taken from Bigand et al. (2005) and one peaceful musical excerpt taken from Eerola and Vuoskoski (2011) were added and used as a debriefing phase. All excerpts had a mean duration of 20 s and were presented as mono files at 16 bits and 44 kHz. Each musical excerpt was normalized to 100% of maximum amplitude.

To ensure that both types of musical emotion (positive high arousing and negative high arousing) did vary significantly in terms of hedonic valence (unpleasant-pleasant) but were similar in terms of arousal (high), we merged the mean scores of valence and energy from a sample of 12 young musicians (20–28 years) reported by Eerola and Vuoskoski (2011) with the mean ratings of valence and arousal we collected in a pilot study from a sample of 30 young no musicians (19–25 years). Two separate ANOVA analyses were performed on the mean ratings of valence and the mean ratings of arousal respectively with the battery (Bigand et al., 2005; Eerola and Vuoskoski, 2011) and the musical emotion (positive high arousing, negative high arousing) as between-subjects factors. Regarding the score of valence, main effects of battery [*1* 79.18, *p* < 0.001; η^2^_*p*_ = 0.80] and musical emotion [*1* 130.12, *p* < 0.001; η^2^_*p*_ = 0.87] as well as a significant interaction between these two factors [*1* 10.51, *p* < 0.05; η ^2^_*p*_ = 0.34] were found. As expected and confirmed by *post-hoc* Bonferroni test comparisons, the mean scores of valence were higher for positive excerpts than for negative excerpts (with *M* = 6.95, *SE* = 0.26 for positive stimuli in Bigand et al.'s study; *M* = 5.03, *SE* = 0.24 for negative stimuli in Bigand et al.'s study; *M* = 5.62, *SE* = 0.24 for positive stimuli in Eerola and Vuoskoski's study; *M* = 2.17, *SE* = 0.24 for negative stimuli in Eerola and Vuoskoski's study). Moreover, the mean scores of valence were significantly higher in the Bigand et al. (2005)'s battery than in the Eerola and Vuoskoski (2011)'s. One possible explanation is that musician listeners tested by Eerola and Vuoskoski were more conservative in their ratings than the non-musician listeners in our pilot study. As expected, no significant mean effect or interaction was found for the arousal ratings, establishing that musical excerpts were well-equalized on this dimension (with *M* = 6.83, *SE* = 0.21 for positive stimuli in Bigand et al.'s study; *M* = 6.67, *SE* = 0.21 for negative stimuli in Bigand et al.'s study; *M* = 6.92, *SE* = 0.21 for positive stimuli in Eerola and Vuoskoski's study; *M* = 6.36, *SE* = 0.21 for negative stimuli in Eerola and Vuoskoski's study).

### Procedure

With the aim of examining how affective and cognitive functioning relate to aspects of expression regulation across age groups, we divided the current experiment into two sessions that were conducted one after the other with the same participants at an interval varying between a few days and 1 month.

In the first session, participants were asked to review and sign a consent form. In accordance with the basic ethical standard, we asked participants to sign a consent form in which they were informed that they were free to withdraw from the study at any time without penalty. For each participant, we ensured that there were not contraindications for using headphones or any skin allergies that would make them sensitive to the electrode gel used in the current research. They were also asked about their age, musical listening skills, education level, self-reported health, visual acuity, and medical history. Auditory perception was controlled using free AudioTest software presenting pure tones at intervals between 500 and 8000 Hz to both ears through a professional 240 Sennheiser headphones. For each participant, the lowest sound pressure level at which each frequency was detected was recorded. Finally, background cognitive and affective measures described above were administered. The first session lasted about 1 h.

In the second session, participants were firstly asked to fill out the State Anxiety Inventory-STAY to assess their emotional state at the moment of the experiment. Afterwards, participants were asked to perform the expression regulation task. The second session lasted about 1 h.

In the emotion regulation task, participants were seated comfortably in front of a computer screen and were instructed to listen attentively to the musical excerpts that were presented binaurally. The loudness of four new musical excerpts intended for training was adjusted subjectively such that the loudness sensation was comfortable for each participant. The experimenter then attached the devices used to measure physiological responses (i.e., skin conductance level, facial EMG, blood pressure, heart rate). A 3-min rest period followed the placement of the electrodes and preceded the onset of the experiment. Participants were asked to remain as still as possible. They began the expression regulation task with the instruction that after each of musical excerpts, they would have to orally report their emotional experience while listening to music. Precisely, they were asked to indicate what they experienced in terms of emotional intensity from 0 “Weak” to 9 “Strong” and hedonic feeling from 0 “Negative” to 9 “Positive” and how much they were engaged in the musical listening from 0 “Not at all” to 9 “Very much.” They were also asked to rate to what extent they tried to apply cognitive reappraisal (also labeled as cognitive control) on the musical event to change their emotional feeling from 0 “Not at all” to 9 “Very much.” The presentation order of these rating scales was counterbalanced across participants. For each rating, the experimenter entered the response on a computer keyboard.

Each participant was assigned to three experimental conditions (i.e., free expression of emotion, expressive enhancement, expressive suppression,) each associated with a specific instruction. The expression regulation task always began with a baseline condition followed by the free expression of emotion condition, which was in its turn followed by the conditions of expressive enhancement and expressive suppression that were both counterbalanced across participants. The experiment ended with a debriefing block of two peaceful musical excerpts for which participants were asked to apply the free expression of emotion instruction. In the baseline condition, four “neutral” auditory excerpts (i.e., orchestra tuning; scales in cello or piano) were presented with the following instruction: “You will listen to auditory excerpts. Be careful because after each excerpt, you will be asked to evaluate what you thought and felt during the listening.” The free expression of emotion instruction was: “You will listen to musical excerpts conveying a positive/negative feeling. We ask you to listen them carefully and to focus on the emotions you feel. While listening to the music, feel the emotions as you want.” The enhancement instruction was (a): “You will listen to musical excerpts conveying a positive/negative feeling. Just pretend you want to share the emotion you experience while listening to the music. To this end, try to express as much as possible your emotion such that someone watching you would clearly know what you are feeling. In other words, while listening to the music, do your best to express as much as possible the emotion you are feeling.” The suppression instruction was: “You will listen to musical excerpts conveying a positive/negative feeling. Just pretend you do not want anything to show what you experience while listening to the music. To this end, try to not express your emotion such that someone watching you would not know that you are feeling anything at all. In other words, while listening to the music, do your best to hide the emotion you are feeling.”

Amongst the 24 musical excerpts selected for the expression regulation task, four positive high arousing stimuli and four negative high arousing stimuli were allocated to the free expression of emotion condition, four positive high arousing stimuli and four negative high arousing stimuli were allocated to the enhancement condition, four positive high arousing stimuli and four negative high arousing stimuli were allocated to the suppression condition. This allocation was counterbalanced across participants. The six Musical Emotion-Expression Instruction pairs were respectively presented in 6 blocks of 4 stimuli from the same musical emotion. The presentation order of these 6 blocks was counterbalanced across participants. Stimulus order within each block was randomized.

For each block of trials, the specific Instruction (i.e., free expression of emotion, expressive enhancement, expressive suppression) was recalled on the center of the screen by a key word (i.e., “LISTEN,” “AMPLIFY,” “SUPPRESS,”) displayed until the participant was ready to begin the trial. The experimenter initiated the trial starting with a baseline period of 12 s of silence during which the participant was instructed to relax. Immediately after, a fixation cross was displayed for 2 s before the presentation of the 20 s musical excerpt. Each musical excerpt was followed by another baseline period of 15 s of silence. After each trial, the set of rating scales (i.e., emotion intensity, hedonic valence, engagement in music listening, cognitive control) was displayed in the center of the screen in a counterbalanced order presentation. The experimenter initiated each trial by pressing a key on a keyboard when the SCL had stabilized. The end of the expression regulation task was immediately followed by another 2-min rest period after which the experimenter removed the physiological measurement sensors.

### Data acquisition and transformation

SCL (μSiemens) was recorded from the left hand, with two Ag–AgCl electrodes attached to the palmar surface of the medial or distal phalanges of the fingers of the non-dominant hand, and filled with isotonic gel. The skin conductance signal was smoothed using the mean of a 1-s moving window. Facial EMG activity (μVolts) was recorded over the left corrugator supercilli and zygomaticus major sites, using two pairs of 4 mm Ag/AgCl shielded electrodes filled with isotonic gel. The EMG data were filtered with a 50 Hz Comb band stop and processed with a root mean square algorithm over 15 samples (with a 30-ms window). ECG data were collected using a simple two leads montage (electrodes on the right arm and left arm, plus a ground on the left leg). The ECG signal was smoothed using the mean of a 3-s moving window. For each participant, heart rate was expressed in beats-per-minute (bpm) for each trial. SBP and DBP (mm/Hg) were measured using noninvasive blood pressure system recorded from the ring and middle fingers of the left hand (brachial artery). They were adjusted continuously to provide an estimate on each heartbeat. SBP and DBP signals were smoothed using the mean of a 3-s moving window. Recording artifacts were visually identified and discarded from the sample. These corresponded to less than 7% of all measurements.

SCL were computed as the difference between the skin conductance signal at the onset of the musical excerpts, which served as a baseline, and the electrodermal activity recorded between 3 s (changes in the form of the tonic electrodermal signal occur 1–4 s after discrete stimuli) and 20 s post-stimulus onset. Facial EMG responses were calculated as the difference between the signal (mean area under the curve per second, μV/s) over the time course of the musical excerpt and a baseline EMG level measured from 1 s prior to the onset of the excerpt (time -1 to 0 s) to the beginning of the excerpt. Given that aging is associated with a reduced physiological flexibility able to produce a prolonged physiological arousal and then a delayed recovery from the event (Charles, 2010), we decided to extract cardiac measures (i.e., HR, SBP, DBP) in two distinct phases of reaction to the musical excerpts: a reactivity period corresponding to the cardiac measurement during music presentation and the recovery period, which is related to the cardiac measurement during the following 15 s of silence after music presentation. For heart rate, the reactivity period was computed as the difference between the mean bpm obtained during the presentation of the excerpts and the mean bpm of the 12 s preceding the excerpt (20 s excerpt −12 s pre-stimulus), whereas the recovery period was calculated by subtracting the mean bpm of the musical excerpt from the mean bpm of the 15 s following the excerpt (15 s post-stimulus −20 s excerpt). Due to technical failures, the data from five participants (3 younger, 2 older) were excluded from the analyses. For SBP and DBP respectively, the reactivity period was computed as the difference between the mean blood pressure obtained during the presentation of the excerpts and the mean blood pressure of the 12 s preceding the excerpt (20 s excerpt −12 s pre-stimulus), whereas the recovery period was calculated by subtracting the mean blood pressure during the musical excerpt from the mean blood pressure of the 15 s following the excerpt (15 s post-stimulus −20 s excerpt). Due to technical failures and as for heart rate measure, the mean blood pressure data from seven participants (i.e., same participants than for BPM analyses plus 1 younger and 1 older adult) were excluded from the analyses. The distribution of each variable (i.e., SCL, facial EMGs, bpm reactivity and bpm recovery, SBP, DBP) was finally examined to identify possible remaining outliers (mean ± 3SD). Based on this criterion, about 0.55% of all measurements were excluded from the analysis.

### Analyses

Data were tested for normal distribution and homogeneity of variance using Shapiro–Wilk and Levene's tests before statistical procedures were applied. Variables for which variance of groups was not equal or that deviated from normality were analyzed with nonparametric statistics using Kruskal–Wallis One-Way ANOVA and Mann–Whitney *U*-tests for between-groups (age) comparisons, as well as Friedman two-way analysis of variance and Wilcoxon signed rank test for within-groups (musical emotions, regulation instruction) comparisons. Student *t*-tests and ANOVAs with age group as between-subject factor and musical emotions (positive, negative) and/or regulation instruction (amplify, suppress) as within-subject factors were applied for data showing a normal distribution and homogeneity of variance.

For each affective (i.e., intensity of emotional experience, hedonic feeling, engagement in musical listening, and self-reported cognitive control), behavioral (i.e., EMG zygomaticus major and corrugator supercilli) and physiological (i.e., SCL, Heart Rate, Blood Pressure) measure, we assessed age equivalence in reactivity to baseline condition (neutral auditory stream). We then evaluated the efficacy of positive and negative musical source of emotions as well as age-related differences in the reactivity to them. The impact of regulation instructions on affective, behavioral and physiological responses was examined depending on musical emotions (positive, negative) and additional analyses were performed to test age-related differences. For each measure, a differential score was calculated to determine the amount of change in response between the regulation instruction condition (amplify or suppress) and the free expression of emotion under the just listen to music instruction. A positive score reflects a greater responsiveness of participants while a negative score reflects a lower responsiveness.

To investigate the consistency between subjective and physiological responses to regulation instructions, correlation analyses were calculated for each experimental condition, separately for younger and older adults. We also examined how the affective and the cognitive functioning relate to expression regulation across age groups. To this end, we computed a set of correlations analyses between sample characteristics and the differential scores of the affective, behavioral, and physiological measures for which age-related differences were found.

## Results

### Age equivalence in baseline condition

Affective (i.e., Intensity of emotional experience, hedonic feeling, engagement in musical listening, and self-reported cognitive control), behavioral (i.e., EMG zygomaticus major and EMG corrugator supercilli) and physiological (i.e., SCL, HR, SBP, DBP) responses to just listen to neutral auditory stimuli were detailed Table 2 for younger and older adults.

**Table 2:** **Age equivalence in baseline condition**.

	**Younger adults**	**Older adults**	**Age comparisons**
	**Mean**	**Mean**	***p*-value^a^^,^^b^**
**AFFECTIVE RESPONSES**			
Intensity (max.09)	3.85 (1.43)	3.33 (1.95)	0.24^a^
Hedonic valence (max.09)	4.34 (1.21)	3.82 (2.00)	0.22^a^
Engagement (max.09)	4.07 (1.57)	3.48 (2.26)	0.23^a^
Reappraisal (max.09)	1.84 (2.12)	2.73 (2.52)	0.20^b^
**BEHAVIORAL RESPONSES**			
EMG zygomaticus major (Area under the curve, mV*sec)	0.0006 (0.0016)	0.0016 (0.0033)	0.23^b^
EMG corrugator supercilli (Area under the curve, mV*sec)	0.0003 (0.0023)	0.0010 (0.0020)	0.12^b^
**PHYSIOLOGICAL RESPONSES**			
SCL (μS)	0.057 (0.139)	0.005 (0.099)	0.26^b^
HR (bpm) reactivity	−2.90 (6.56)	3.18 (6.82)	0.00^b^
SBP (mmHg) reactivity	0.41 (1.86)	0.35 (2.45)	0.92^a^
DBP (mmHg) reactivity	0.34 (1.88)	1.61 (2.09)	0.03^b^
HR (bpm) recovery	−4.07 (7.17)	0.52 (5.93)	0.01^b^
SBP (mmHg) recovery	0.31 (1.82)	−0.55 (1.68)	0.08^a^
DBP (mmHg) recovery	−0.38 (1.81)	0.42 (1.92)	0.11^a^

*Standard deviations are listed in parentheses; SCL, Skin Conductance Level; HR, Heart Rate; SBP, Systolic Blood Pressure*.

a *Comparisons were assessed by Student's t-test*.

b *Comparisons were assessed by Mann–Whitney's U-test*.

#### Affective responses

No significant main effect of age group was found for any of the intensity of emotional experience, hedonic feeling, and engagement in musical listening or self-reported cognitive control ratings.

#### Behavioral responses

There was no significant main effect of age group on facial EMG, neither for zygomaticus major, nor for corrugator supercilli activity.

#### Physiological responses

We found a main effect of age group indicating that compared to younger adults, older adults' HR and DBP was slower during reactivity period (with respectively, *p* < 0.001, Mann–Whitney *U* = 192 and *p* = 0.03, Mann–Whitney *U* = 240). This age effect was also observed in recovery period for HR (*p* = 0.01, Mann–Whitney *U* = 237) but not for DBP [*t*_(52)_ = −1.59, *p* = 0.12]. No significant main effect of age group on SBP was found neither during reactivity period nor during recovery period.

### Free expression of emotion under the just listen to music instruction

Affective, behavioral, and physiological responses to positive and negative musical emotions were detailed for each age group Table 3.

**Table 3:** **Spontaneous responses to just listening to musical emotions in younger and older adults**.

	**Younger adults**		**Older adults**		**Age comparisons, *p*-value^a^^,^^b^**	
	**Positive music**	**Negative music**	**Positive music**	**Negative music**	**Positive music**	**Negative music**
**AFFECTIVE RESPONSES**						
Intensity (max.09)	5.40 (1.57)	5.64 (1.47)	6.03 (1.43)	4.90 (1.68)	0.12^b^	0.04^b^
Hedonic valence (max.09)	6.35 (1.10)	4.20 (1.53)	6.13 (1.65)	4.63 (1.96)	0.71^b^)	0.35^b^
Engagement (max.09)	5.75 (1.66)	5.68 (1.64)	6.41 (1.35)	5.21 (1.73)	0.09^a^	0.28^a^
Reappraisal (max.09)	1.51 (1.92)	1.75 (2.16)	3.06 (2.76)	2.81 (2.53)	0.04^b^	0.10^b^
**BEHAVIORAL RESPONSES**						
EMG zygomaticus major (Area under the curve, mV*sec)	0.001 (0.002)	0.000 (0.001)	0.002 (0.004)	0.001 (0.001)	0.58^b^	0.12^b^
EMG corrugator supercilli (Area under the curve, mV*sec)	0.001 (0.002)	0.002 (0.009)	0.001 (0.002)	0.001 (0.002)	0.17^b^	0.16^b^
**PHYSIOLOGICAL RESPONSES**						
SCL (μS)	0.016 (0.11)	−0.007 (0.12)	0.037 (0.08)	−0.009 (0.08)	0.26^b^	0.87^b^
HR (bpm) reactivity	0.36 (7.26)	−0.20 (6.07)	−1.05 (5.67)	2.36 (5.53)	0.25^b^	0.12^b^
SBP (mmHg) Reactivity	0.47 (1.59)	1.30 (2.63)	1.21 (2.08)	0.63 (1.73)	0.10^b^	0.65^b^
DBP (mmHg) reactivity	1.37 (1.62)	0.79 (2.07)	0.96 (1.79)	1.48 (1.43)	0.39^a^	0.16^a^
HR (bpm) recovery	−2.49 (7.67)	−1.57 (9.63)	−0.143 (1.50)	1.632 (1.53)	0.27^b^	0.10^b^
SBP (mmHg) recovery	0.00 (1.70)	0.52 (2.55)	0.34 (2.41)	−0.89 (1.60)	1.00^b^	0.06^b^
DBP (mmHg) recovery	0.38 (1.66)	0.29 (2.35)	0.21 (2.18)	0.21 (1.00)	0.92^b^	0.97^b^

*Standard deviations are listed in parentheses; EMG, Electromyography; SCL, Skin Conductance Level; HR, Heart Rate; SBP, Systolic Blood Pressure; DBP, Diastolic Blood Pressure*.

a *Comparisons were assessed by follow-up simple planned contrats on the within-factor Emotion Category (Positive vs. Negative music) previously entered into a Manova with Age-Group (Younger vs. Older) as within-factor*.

b *Comparisons were assessed by the non parametric Mann–Whitney's U-test*.

#### Efficacy of musical emotions

A first set of analyses was conducted to test whether participants' responses did change as a function of the musical emotion (positive, negative).

***Affective responses***. As expected, results indicated that participants judged the hedonic valence of their emotional state more positive (Wilcoxon Signed Ranks, *Z* = 5.58, *p* = 0.000) during positive music than during negative music. Results also indicated that participants reported to be greater engaged with music listening [*F*_(1, 59)_ = 9.67, *p* = 0.003; η^2^_*p*_ = 0.14] during positive than during negative musical excerpts.

***Behavioral responses***. In line with the results above, participants displayed greater EMG zygomaticus major activity (Wilcoxon Signed Ranks, *Z* = 2.95, *p* = 0.003) during positive music than during negative music.

***Physiological responses***. In agreement with the affective responses, participants showed higher SCL (Wilcoxon Signed Ranks, *Z* = 2.33, *p* = 0.020) during positive than during negative musical excerpts. No other significant main effect of musical emotion was observed.

#### Age-related differences

***Affective responses***. Older adults felt less emotional intensity than younger adults while listening to negative music (*p* = 0.04, Mann–Whitney *U* = 325) and reported to apply greater cognitive control during positive music (*p* = 0.04, Mann–Whitney *U* = 323).

***Behavioral responses***. No significant main effect of age group was found.

***Physiological responses***. There was significant main effect of age group.

### Effect of regulation instructions

Affective, behavioral, and physiological responses to positive and negative musical emotions as a function of the regulation instructions (amplify, suppress) and age group were detailed Table 4.

**Table 4:** **Affective, behavioral and physiological responses to regulation instructions as a function of musical emotions in younger and older adults**.

	**Younger adults**				**Older adults**				**Age comparisons, *p*-value**			
	**Positive music**		**Negative music**		**Positive music**		**Negative music**		**Positive music**		**Negative music**	
	**Amplify**	**Suppress**	**Amplify**	**Suppress**	**Amplify**	**Suppress**	**Amplify**	**Suppress**	**Amplify**	**Suppress**	**Amplify**	**Suppress**
**AFFECTIVE RESPONSES**												
Intensity (max.09)	5.42 (1.43)	5.41 (1.56)	5.19 (1.67)	4.73 (1.57)	6.06 (1.59)	5.73 (1.58)	5.07 (1.72)	4.62 (1.81)	0.73	0.63	0.07	0.02
Hedonic Valence (max.09)	6.63 (1.11)	6.40 (1.09)	3.27 (1.25)	3.49 (1.24)	6.45 (1.41)	6.01 (1.58)	4.38 (1.59)	4.41 (1.73)	0.93	0.44	0.14	0.27
Engagement (max.09)	5.65 (1.77)	5.62 (1.75)	5.22 (2.04)	5.11 (1.85)	6.50 (1.80)	5.38 (1.58)	5.11 (1.77)	4.71 (1.76)	0.38	0.26	0.29	0.53
Reappraisal (max.09)	4.48 (2.51)	4.69 (2.75)	4.23 (2.67)	4.89 (2.57)	4.72 (2.57)	5.79 (2.65)	4.68 (2.38)	5.47 (2.57)	0.02	0.37	0.40	0.44
**BEHAVIORAL RESPONSES**												
EMG zygomaticus major (Area under the curve, mV*sec)	0.005 (0.004)	0.001 (0.002)	0.001 (0.002)	0.000 (0.001)	0.005 (0.007)	0.001 (0.003)	0.004 (0.005)	0.000 (0.004)	0.13	0.87	0.14	0.99
EMG corrugator supercilli (Area under the curve, mV*sec)	0.003 (0.007)	0.001 (0.005)	0.010 (0.011)	0.001 (0.004)	0.003 (0.004)	0.001 (0.002)	0.009 (0.013)	0.001 (0.002)	0.87	0.33	0.32	0.46
**PHYSIOLOGICAL RESPONSES**												
SCL (μS)	0.021 (0.18)	−0.059 (0.18)	0.014 (0.14)	−0.058 (0.11)	0.026 (0.12)	−0.013 (0.05)	0.020 (0.11)	−0.036 (0.08)	0.86	0.41	0.71	0.49
HR (bpm) reactivity	1.47 (7.13)	−2.22 (5.33)	0.39 (7.16)	−1.09 (5.67)	1.16 (6.49)	−1.77 (5.25)	0.17 (4.91)	−0.89 (5.16)	0.33	0.29	0.08	0.47
SBP (mmHg) reactivity	0.77 (2.92)	0.74 (1.76)	0.83 (2.33)	0.38 (2.67)	1.04 (2.42)	0.92 (1.68)	1.05 (2.04)	0.48 (2.46)	0.15	0.48	0.25	0.43
DBP (mmHg) reactivity	1.69 (2.27)	0.74 (1.59)	1.04 (2.21)	0.46 (2.95)	1.29 (1.96)	0.58 (1.70)	1.17 (1.64)	0.59 (2.03)	0.45	0.32	0.49	0.51
HR (bpm) recovery	2.74 (12.92)	−4.19 (6.16)	1.03 (8.81)	−1.69 (4.97)	1.24 (6.42)	−2.01 (7.15)	−1.03 (6.26)	−2.73 (7.20)	0.33	0.71	0.13	0.10
SBP (mmHg) recovery	−0.39 (4.57)	−0.36 (2.49)	0.32 (2.71)	−0.06 (1.91)	−0.02 (2.03)	−0.37 (2.30)	0.32 (2.51)	0.01 (1.43)	0.52	0.63	0.15	0.01
DBP (mmHg) recovery	0.32 (2.91)	−0.30 (2.00)	1.76 (2.75)	0.29 (1.91)	0.16 (2.09)	−0.23 (2.34)	0.40 (2.12)	−0.03 (2.12)	0.66	0.95	0.07	0.53

*Standard deviations are listed in parentheses; EMG, Electromyography; SCL, Skin Conductance Level; HR, Heart Rate; SBP, Systolic Blood Pressure; DBP, Diastolic Blood Pressure. Comparisons were assessed by non parametric comparisons (Mann–Whithney U and pairwise Wilcoxon signed-rank tests) conducted on differential scores derived by subtracting spontaneous listening condition from the amplify and suppress conditions, respectively*.

#### Regulation instructions: manipulation check

In order to test participants' ability to implement the regulation instructions, we conducted nonparametric Friedman ANOVAs separately on the differential scores of EMG zygomaticus major activity and EMG corrugator supercilli activity. For each analysis, the four experimental conditions (expressive suppression during positive music, expressive amplification during positive music, expressive suppression during negative music, expressive amplification during negative music) were entered as within-subjects factor.

Statistically significant changes were found across experimental conditions both for the EMG zygomaticus major activity and the EMG corrugator supercilli activity (Friedman's ANOVAs, *p* < 0.001). As expected, follow-up Wilcoxon signed-rank tests showed that participants displayed greater EMG zygomaticus major activity when instructed to amplify their expression than when instructed to suppress it during positive music (Wilcoxon Signed Ranks, *Z* = 5.73, *p* = 0.000). Moreover, they showed greater EMG corrugator supercilli activity when they were instructed to amplify their expression than when they were instructed to suppress it during negative music (Wilcoxon Signed Ranks, *Z* = 6.16, *p* = 0.000).

#### Age differences in regulated behavior

***Affective responses***. A set of Mann–Whitney *U*-tests indicated that compared to their younger counterparts, older adults reported experiencing higher emotional intensity (*p* = 0.02, Mann–Whitney *U* = 306) when they were instructed to suppress their expression during negative music. Results also indicated that older adults reported to apply lesser cognitive control to change their feeling than their younger counterparts when they were instructed to amplify their expression during positive music (*p* = 0.02, Mann–Whitney *U* = 304).

***Behavioral responses***. There was no effect of age group on differential scores of EMG zygomaticus major activity or on EMG corrugator supercilli activity, whatever the expressive regulation condition (amplification, suppression) or the musical excerpt (positive, negative). Additional *t*-test comparisons to a standard 0 indicated that older adults showed a significantly reduced EMG zygomaticus major activity in condition where they were instructed to suppress their expression during positive music [*t*_(31)_ = −2.16, *p* < 0.05], whereas the reduced EMG zygomaticus major activity observed in younger adults almost reached significance [*t*_(30)_ = −1.76, *p* = 0.08].

***Physiological responses***. In congruence with their affective responses, older adults showed higher SBP in recovery period (*p* = 0.01, Mann–Whitney *U* = 213) than the younger adults in condition of expressive suppression during negative music.

For each age group, additional one-sample *t*-tests were computed to verify whether the mean value of the differential scores for which age-related differences were found (i.e., intensity of emotional experience, SBP, and self-reported cognitive control) were significantly different from 0. In the condition where participants were instructed to suppress expression while listening to negative music, the differential score of emotional intensity was significantly lower from 0 in younger [*t*_(30)_ = −4.74, *p* < 0.001] but not in older adults [*t*_(29)_ = −0.93, *p* = 0.36] while the differential score of SBP was significantly higher from 0 in older [*t*_(29)_ = 3.21, *p* = 0.003] but not in younger adults [*t*_(30)_ = −1.06, *p* = 0.29]. Consistently with the findings above, this indicates that compared to the just listening condition, the suppression of negative expression globally produced higher emotional activation in older but not in younger adults. Regarding the self-reported cognitive control, results indicated that, in condition where participants were instructed to amplify expression while listening to positive music, the differential score was significantly higher from 0 both in younger [*t*_(30)_ = 7.06, *p* < 0.001] and older [*t*_(29)_ = 4.30, *p* < 0.001] adults, indicating that compared to the just listening condition, the enhancement of positive expression produces higher cognitive control both in younger and older adults. However, and as mentioned above, younger adults reported a higher level of cognitive control than older adults.

### Relationship between affective and physiological responses in younger and older adults

In order to examine the consistency between participants' subjective assessment and their autonomic reaction in response to the regulation instructions, we conducted additional correlation analyses between the differential scores of affective responses (i.e., Intensity of emotional experience, hedonic feeling, engagement in musical listening, and self-reported cognitive control), and the differential scores for physiological responses (i.e., SCL, HR, SBP, DBP) to each experimental condition, separately for younger and older adults. A Bonferroni correction was used to account for the inflated chance of a Type I error associated with conducting multiple correlations. For each experimental condition (expressive amplification while listening to positive music, expressive suppression while listening to positive music, expression amplification while listening to negative music, expression suppression while listening to positive music), we adjusted the α level from 0.05 to 0.05 divided by 28 (4 affective measures by 7 physiological indexes), or 0.002, meaning that we only considered comparisons with *p* = 0.002 or less to be significantly different. In this respect, no significant correlation was found between subjective rating and physiological responses under the regulation instructions.

### Relationship between sample characteristics and responses to regulation instructions

Because further analyses indicated a significant or an almost significant difference between age groups for classical music listening habit, fluid intelligence (Raven Matrices), cognitive flexibility (duration for TMT execution), suppression strategy (ERQ), agreeableness (score A on NEO-P-IR), conscientiousness (score C on NEO-P-IR), and auditory thresholds for all of the five frequencies, these factors were introduced in separate correlation analyses to estimate their linkage with the differential scores for which we found age-related differences (i.e., Intensity of emotional experience in suppression condition during negative music, cognitive control in amplification condition during positive music, SBP in suppression condition during negative music). Results showed that SBP recorded in suppression condition during negative music was positively and significantly correlated with cognitive flexibility, *r*_(52)_ = 0.27, *p* < 0.05, and auditory thresholds at 1000 Hz, *r*_(52)_ = 0.35, *p* < 0.05 and 8000 Hz, *r*_(52)_ = 0.27, *p* < 0.05, explaining 7, 12, and 7% of the variance, respectively. No other significant relationship with sample characteristics was found neither for the intensity of emotional experience nor for the cognitive control rating.

## Discussion

The purpose of this study was to examine the effects of two response-focused regulation strategies (i.e., expressive enhancement, expressive suppression) following the exposure to positive and negative musical emotions on affective, behavioral, and physiological parameters in younger and older adults. The control for the age equivalence in the condition where participants had to listen to a neutral auditory stream indicated that older adults reported affective and showed behavioral responses that were similar to those of their younger counterparts. This indicates that the age-related differences described above for affective and behavioral responses were linked to the emotion felt during positive and negative music and were not due to fundamental subjective or physical differences between younger and older adults. Regarding physiological responses, our findings indicated that compared to younger adults, older adults showed decreased heart rate and blood pressure in response to neutral auditory stream. This is consistent with notion that auditory stream may have beneficial effects on blood pressure and heart rate (Guzzetta, 1989; Chafin et al., 2004), especially in people with mild hypertension such as the elderly.

Controlling for the behavioral effect of the regulation instruction whatever the age, we found that the behavioral responses indexed by the facial EMG activity did significantly vary as a function of the instruction. The direction of these changes verified that participants succeed to implement expressive suppression and amplification. More especially, and as expected, we observed that older adults were as efficient as younger adults to exaggerate or inhibit their facial expressions. This corroborates previous data providing evidence for the preservation of older adults' ability to implement behavioral regulation (Kunzmann et al., 2005; Phillips et al., 2008; Henry et al., 2009; Shiota and Levenson, 2009). This also supports the view that aging is not always synonymous with decline in expressiveness (Levenson et al., 1991; Tsai et al., 2000).

In the condition where participants were instructed to suppress expression elicited by positive music, no significant age-related difference was observed. On the contrary, when instructed to suppress expression elicited by negative music, older adults reported experiencing higher emotional intensity and showed higher SBP during the recovery period than younger adults. This suggests that the suppression of negative expression was more cognitively and physiologically demanding for older adults than for younger adults. More generally, these findings neither show that if older adult prioritize the processing of positive stimuli, they should have more difficulties to inhibit the related expressions, nor support the idea that older adults are better than younger adults to regulate negative emotions. Instead, they corroborate the view that, due to decreased cognitive resources and dysregulation in multiple physiological systems (Charles, 2010), older adults would be more vulnerable to stressors than younger adults (Labouvie-Vief, 2008). These data also converge nicely with and extend the previous work of Opitz et al. (2012) demonstrating that in condition when gaze direction is controlled (preventing strategic avoidance of unpleasant scenes), older adults were less successful than younger adults to decrease unpleasant emotion elicited by visual stimuli. Furthermore, the current results dispute the idea that the intensity of the emotion felt may decline with age. They are also compatible with the hypothesis that, in a condition that prevents attention redirection, expressive suppression has stronger affective and physiological impact on older than on younger adults' responses. Thus, they support the hypothesis of age-related difficulties in deliberately suppressing behaviors (e.g., Kramer et al., 1994). Importantly, current findings contrast with previous results showing that, for the expressive suppression condition and in comparison with the condition of just listen to music, older adults displayed as less negative affect as their younger counterparts (Kunzmann et al., 2005; Phillips et al., 2008). One explanation could be that previous experiments were based on visual emotion elicitors against which gaze redirection may be used to avoid and thus to reduce the impact of emotional stimuli. Consistently with this hypothesis, Phillips et al. (2008) observed that older adults showed more gaze aversion than younger adults in suppression than in spontaneous expression condition. In the current study, when faced with negative source of emotion from which it is not possible to redirect attention, age-related differences emerge on affective and physiological responses. Accompanying these findings, we showed that older adults paradoxically reported using more expressive suppression and less cognitive reappraisal in daily life (i.e., Emotion Regulation Questionnaire) than their younger counterparts. Although in line with recent findings showing that older adults make greater use of suppression strategy (Nolen-Hoeksema and Aldao, 2011; Brummer et al., 2013), our data highlight the potential disconnection between what people reported on their emotion regulation skills and what they actually did. Thus, it will be useful to conduct additional experiments to give more insight into this inconsistency.

In the condition where participants were instructed to enhance expression elicited by negative music, no significant age-related difference was observed However, our results showed an age-related difference indicating that the instruction to enhance positive expression seems to require lesser cognitive control in older than in younger adults. Although this result was based on self-report and cannot provide definitive evidence, it raises the question whether the behavioral enhancement of the already-initiated positive expression may be less cognitively costly in older adults. Further investigation is needed to highlight this question.

When asked to just listen to negative music, older adults reported experiencing less negative emotion than younger adults while in the condition where the instruction was to just listen to positive music, older adults reported greater cognitive control in comparison with their younger counterparts. Taken together, these data indicate that aging is associated with a reduction of negative experience and a greater investment in emotion regulation. These results did not strictly reflect a bias toward positive information but they are compatible with the hypothesis that as people age, and in the absence of explicit instruction to regulate emotion, they are geared toward the maintenance of well-being. This is in line with previous findings indicating that when the experimental condition allows emotional goals to not be constrained by cognitively demanding instructions, the positivity effects are most likely to be observed (e.g., Kennedy et al., 2004; Knight et al., 2007; Yang and Ornstein, 2011; Tomaszczyk and Fernandes, 2012). More generally, these findings are consistent with previous findings showing age-related changes in the way musical emotions are appraised (Vieillard et al., 2012; Vieillard and Gilet, 2013; Vieillard and Bigand, 2014).

Taken into account the prediction that the positivity effect is a product of age differences in default motivational priorities as opposed to information processing abilities, it could be logically argued that the age-related difficulty in the ability to deliberately suppress negative emotions is compatible with findings and *Socioemotional Selectivity Theory* on positivity effect. Nevertheless, this view supposes the existence of a causal link between older adults' preference for positive over negative information and emotion regulation outcomes that has not yet been convincingly demonstrated (Isaacowitz and Blanchard-Fields, 2012; Reed and Carstensen, 2012). It is worthy to note that the hypothesis of motivational goals constraints should lead to the prediction that older adults' preference or motivational goals would vanish, then producing equivalent regulation skills between age groups. However, this is not what we found. At this stage, we argue that our findings are consistent with the idea that emotion regulation abilities suffer from age-related difficulties to suppress negative emotions elicited by emotional stimuli that do not allow perceptual attention to be redirected away from the emotional source. In the future, further investigations are needed to examine the link between positivity in emotional processing and emotion regulation outcomes in order to highlight the conditions in which motivational goals constraints may affect emotion regulatory abilities in aging.

A caveat would be that the lack of pilot study ensuring equivalent emotional ratings across age groups could explain age-related differences observed in emotion regulation skills. However, the fact that the negatively valenced musical stimuli were repeatedly found to elicit less intense emotional experience in older adults than in younger adults (Vieillard et al., 2012; Vieillard and Gilet, 2013; Vieillard and Bigand, 2014) across different set of musical stimuli is a result *per se*. Moreover, it is consistent with prior findings indicating that older adults demonstrated lower arousal ratings of negative pictures compared to younger counterpart (e.g., Mather et al., 2004). Such findings suggest an age-related change in the way negative stimuli is experienced. More importantly, the selection of the musical stimuli cannot explain why the decrease of negative experience in older adults co-occurs with a lesser ability to down-regulate the negative expression of emotion. Instead, and as outlined above, it is possible that in the absence of explicit instruction to regulate the expression of their emotion, older adults are, by default, engaged in emotional processing that favor the reduction of the subjective emotional perception of negative events. This is in line with prior investigations demonstrating that compared to younger adults, older adults show reduced responses selectively to negative stimuli (Mather et al., 2004) and decreased amygdala activity for the trials in which they reported experiencing negative pictures as more neutral (St Jacques et al., 2010). On the opposite, in condition where the instructions are to inhibit the expression of emotion, and given that the suppression instruction is known to be more cognitively and physiologically costly than the simply listening condition (Gross, 1998), it is possible that the effort required by this resource-demanding task makes older adults less capable to maintain a low level of physiological activation than their younger counterparts. This is in line with the idea that when faced with unpleasant source of emotion, the implementation of cognitively costly task like expressive suppression may become synonymous with a disturbing situation, in particular in older adults who suffered from reduced cognitive resources (Labouvie-Vief, 2008).

One potential limitation of our study is that our sample sizes were quite small. On the other hand, it could be argued that the relatively broad age ranges may increase the generalizability of our results and thus constitute a methodological strength. However, the potential implication is that this might create greater variability in each age group and subsequently drop the age-related differences in emotional responses. Therefore, further investigations are needed with larger sample size to extend our findings. It could also be argued that the age-related difference (that almost reached the threshold of statistical significance) in the frequency of classical music listening could have influenced our current results. One way to estimate how much this age-related difference may have influenced the way the younger and the older listeners implement the expressive regulation instruction is by verifying whether both age groups were differently engaged in the musical listening. Our findings clearly showed that they were not, suggesting that even though older adults in our sample listen to classical music much more frequently, this difference had no strong influence on the way younger and older listeners were engaged in the musical listening and probably neither on the way they regulated the expression of emotions. Another potential limitation is related to the form of emotion regulation investigated. As stated above, we choose to examine only one kind of emotion regulation strategy: the expressive regulation of emotion in response to music. To provide a complete picture of emotion regulation in older adults, future researches have to address other forms of regulation such as those based on cognitive reappraisal. We are currently involved in the investigation of the age-related differences in affective, behavioral, physiological, and cognitive consequences of emotion regulation based on positive and detached reappraisal. The aim is to test whether previous findings indicating age-related decline in ability to implement detached reappraisal, and age-related enhancement of ability to implement positive reappraisal (Shiota and Levenson, 2009) were confirmed in the context of musical emotions. Within these limitations, however, this study has provided new insight suggesting that in contrast with the notion that emotion regulation represents a domain of human functioning that would improve with older age, the expressive regulation of negative emotion might suffer from age-related losses.

### Conflict of interest statement

The authors declare that the research was conducted in the absence of any commercial or financial relationships that could be construed as a potential conflict of interest.
